# An *Ab Initio* Study of Thermodynamic and Mechanical Stability of Heusler-Based Fe_2_AlCo Polymorphs

**DOI:** 10.3390/ma11091543

**Published:** 2018-08-27

**Authors:** Martin Friák, Sabina Oweisová, Jana Pavlů, David Holec, Mojmír Šob

**Affiliations:** 1 Institute of Physics of Materials, Academy of Sciences of the Czech Republic, Žižkova 22, CZ-616 62 Brno, Czech Republic; 2 Department of Chemistry, Faculty of Science, Masaryk University, Kotlářská 2, CZ-611 37 Brno, Czech Republic; 394665@mail.muni.cz; 3 Central European Institute of Technology, CEITEC MU, Masaryk University, Kamenice 5, CZ-625 00 Brno, Czech Republic; houserova@chemi.muni.cz (J.P.); mojmir@ipm.cz (M.Š.); 4 Department of Physical Metallurgy and Materials Testing, Franz-Josef-Strasse 18, A-8700 Leoben, Austria; david.holec@unileoben.ac.at

**Keywords:** CoAlFe_2_, AlCoFe_2_, Fe_2_CoAl, AlFe_2_Co, Heusler, disorder, *ab initio*, stability, elasticity

## Abstract

We use quantum-mechanical calculations to test a hypothesis of Glover et al. (J. Mag. Mag. Mater. 15 (1980) 699) that Co atoms in the Fe${}_{2}$AlCo compound have on average 3 Fe and 3 Co atoms in their second nearest neighbor shell. We have simulated four structural configurations of Fe${}_{2}$AlCo including the full Heusler structure, inverse Heusler polymorph and two other phases matching this idea. The highest thermodynamic stability at *T* = 0 K is indeed predicted for one of the phases with the distribution of atoms according to Glover and et al. However, small energy differences among three of the studied polymorphs lead to a disordered CsCl-structure-like (B2-like) phase at elevated temperatures. The fourth variant, the full Heusler phase, is predicted to be mechanically unstable. The global magnetic states are predicted to be ferromagnetic but local magnetic moments of Fe and Co atoms sensitively depend on the composition of the first and second coordination shells.

## 1. Introduction

Ternary compounds with the chemical formula X${}_{2}$YZ and one of Heusler-type structures [1] are a very rich family of materials. They have been very intensively studied [2,3], including their magnetic properties [4,5,6], half-metallic features [7,8,9,10,11,12], magneto-optical properties [13], semiconductor-like electrical resistivity [14], thermoelectric aspects [15,16], topological quantum properties [17,18] or shape-memory features [19,20,21] among many others.

**Figure 1:**
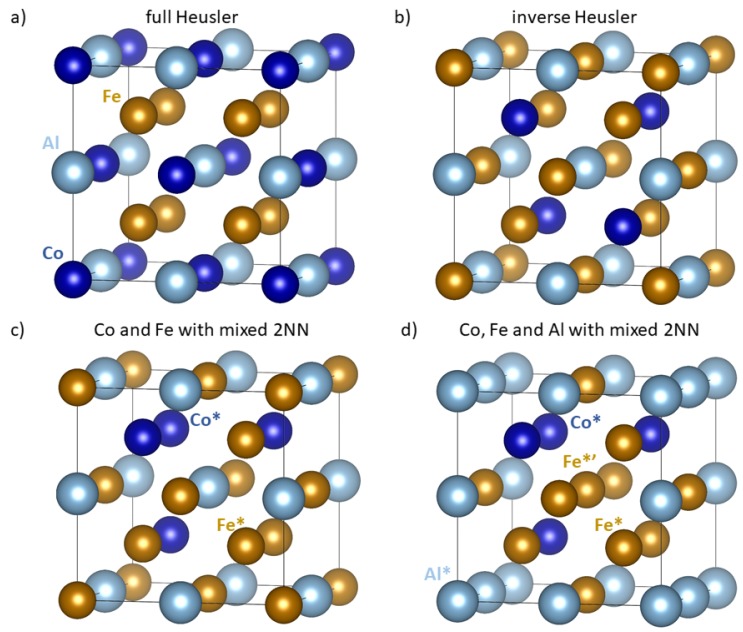
Schematic visualizations of 16-atom supercells of Fe${}_{2}$AlCo polymorphs used in our quantum-mechanical calculations (some atoms are shown with their periodic images). (**a**) Full Heusler structure. (**b**) Inverse Heusler structure. (**c**) Specific distribution of Co (and Fe) atoms regarding the atoms in the 2NN shell (see the text). (**d**) Special arrangement of atoms of all three chemical species. Selected atoms discussed in the text are marked by a star and/or a prime symbol.

As far as theoretical studies are concerned, Gilleßen and Dronskowski published recently two very thorough papers covering 810 different ternary compounds and tested their thermodynamic, structural and magnetic properties in the case of the full Heusler structure [22] (see Figure 1a) or the inverse Heusler structure [23] (see Figure 1b). Using the schematic chemical formula X${}_{2}$YZ mentioned above, they considered X representing the elements Fe, Co, Ni, Ru, Rh, Pd, Os, Ir, and Pt, then Y standing for the elements Sc, Ti, V, Cr, Mn, Fe, Co, Ni, Cu, and Zn, and Z representing the atoms of Al, Ga, In, Tl, Ge, Sn, Pb, Sb, and Bi. These two papers covered also the Fe${}_{2}$AlCo compound and Gilleßen and Dronskowski showed that Fe${}_{2}$AlCo in the inverse Heusler structure has a lower formation energy, possesses magnetic moment and does not tetragonally deform, for details see Ref. [23]. It is important to note that regarding the two structural types studied by Gilleßen and Dronskowski, Co atoms have in the second nearest neighbor (2NN) coordination shell either 6 Al atoms (full Heusler structure) or 6 Fe atoms (inverse Heusler structure) within these Fe${}_{2}$AlCo crystals.

Our study was motivated by experimental paper by Glover et al. [24] where a different composition of the second nearest neighbor coordination shell of Co atoms was proposed. Glover et al. combined X-ray and Moessbauer spectroscopy techniques and suggested that Co atoms have on average 3 Fe and 3 Co atoms in their second nearest neighbor shell. Our paper is thus focused on testing this hypothesis employing state-of-the-art quantum-mechanical calculations.

## 2. Materials and Methods

Our quantum-mechanical calculations of Fe${}_{2}$AlCo polymorphs were performed using the Vienna Ab initio Simulation Package (VASP) [25,26] which implements the density functional theory [27,28] and projector augmented wave potentials [29,30]. The exchange and correlation energy was treated in the generalized gradient approximation (GGA) as parametrized by Perdew and Wang [31] using the Vosko-Wilk-Nusair correction [32]. We used a plane-wave energy cut-off of 350 eV with a 10 × 10 × 10 Monkhorst-Pack k-point mesh in the case of 16-atom supercells (see Figure 1).

We studied four polymorphs including the full Heusler structure (Figure 1a) and the inverse Heusler phase (Figure 1b). Next, we also calculated properties of two other polymorphs (Figure 1c,d) which have the sublattice containing Co atoms arranged in a very specific manner. In particular, one Co atom (see the Co* atom in Figure 1c,d) has only Co atoms in the 2NN coordination shell and the other three Co atoms have 2 Co and 4 Fe atoms in the 2NN coordination sphere. The average number of atoms in the 2NN sphere of Co atoms is thus 3 Co and 3 Fe, i.e., numbers equal to those suggested by Glover et al. [24]. The same is true for Fe atoms on this sublattice (see the atom Fe* with 6 Fe atoms in the 2NN shell). In the case of the polymorph visualized in Figure 1c, the sublattice containing solely Fe and Al atoms is the same as in the inverse Heusler structure is Figure 1b. The last studied polymorph (Figure 1d) has the sublattice containing only Fe and Al atoms rearranged so that both Al and Fe atoms contain on average 3 Fe and 3 Al atoms in their 2NN shell (see the atom Al* with 6 Al atoms in the 2NN shell and the atom marked as Fe*’ with 6 Fe atoms in the 2NN shell in Figure 1d).

The thermodynamic stability was analyzed by evaluating the formation energy $E_{f}$ (per atom). For a crystal ${\mathrm{Fe}}_{\alpha }{\mathrm{Al}}_{\beta }{\mathrm{Co}}_{\gamma }$ containing α atoms of Fe, β atoms of Al and γ atoms of Co, it is defined as

(1) $$E_{f}\left({\mathrm{Fe}}_{\alpha }{\mathrm{Al}}_{\beta }{\mathrm{Co}}_{\gamma }\right)=\left(E\left({\mathrm{Fe}}_{\alpha }{\mathrm{Al}}_{\beta }{\mathrm{Co}}_{\gamma }\right)-\alpha \mu^{\mathrm{Fe}}-\beta \mu^{\mathrm{Al}}-\gamma \mu^{\mathrm{Co}}\right)/\left(\alpha +\beta +\gamma \right)$$

where $\mu^{\mathrm{Fe}}$, $\mu^{\mathrm{Al}}$, and $\mu^{\mathrm{Co}}$ are chemical potentials of the constituents which we set equal to the energy of ferromagnetic (FM) body-centered cubic (bcc) Fe, non-magnetic (NM) face-centered cubic (fcc) Al and FM hexagonal close-packed (hcp) Co, respectively. These energies were computed using 2-atom cells in the case of Fe and Co, and a single-atom cell in the case of Al. We employed the same cut-off energy as listed above and 20 × 20 × 20, 24 × 24 × 15 and 24 × 24 × 24 k-point meshes in the case of elemental FM bcc Fe, FM hcp Co and NM fcc Al, respectively. The mechanical stability of Fe${}_{2}$AlCo polymorphs was assessed by computing a full tensor of single-crystal elastic stiffnesses. These were determined employing the stress-strain method [33]. When any eigenvalue of the elastic-stiffnesses matrix reaches a negative value, i.e., a condition of elastic stability is not satisfied [34], the crystal is unstable.

## 3. Results

Calculated properties of the four studied polymorphs of Fe${}_{2}$AlCo are summarized in Table 1. Considering the formation energy, our values for the full and inverse Heusler structures, –0.145 and –0.260 eV/atom, are in an excellent agreement with the values of –0.155 and –0.271 eV/atom published by Gilleßen and Dronskowski [23]. The difference of about 0.01 eV/atom between our results and those from Ref. [23] is most likely caused by different energy cut-off plane wave expansion (500 eV) and k-point mesh (8 × 8 × 8) used by Gilleßen and Dronskowski. Both sets of calculated results neatly agree in the case of lattice parameters and magnetic moments. In particular, our predicted equilibrium lattice parameter $a_{\mathrm{eq}}$ = 5.697 Å for the inverse Heusler is nearly identical to the value 5.701 Å published by Gilleßen and Dronskowski. Both values are also very close to the experimental values 5.75 Å [24].

Our predicted total magnetic moment of the full Heusler polymorph, $\mu^{\mathrm{TOT}}$ = 5.70 $\mu_{B}$ per formula unit (f.u.), agrees with the value 5.66 $\mu_{B}$/f.u. published in Ref. [22]. Even better agreement is obtained in the case of the inverse Heusler polymorph when we predict the total magnetic moment 4.99(8) $\mu_{B}$/f.u. and 5.00 $\mu_{B}$/f.u. was reported in Ref. [23]. All these values are also quite close to the experimental room-temperature magnetic moment of 4.4 $\mu_{B}$/f.u. published in Ref. [24] (the computed *T* = 0 K values are comparable with the room-temperature experimental ones as Curie temperature is 1010 K [35]).

After cross-checking our calculations with earlier published results and proving their reliability, let us focus on the two polymorphs with specific arrangements of atoms (with 3 Co and 3 Fe atoms in the 2NN shell of Co atoms). The formation energies in Table 1 show that the structural configuration shown in Figure 1c, which has the sublattice containing Fe and Al as in the inverse Heusler structure, exhibits the most negative formation energy (the highest thermodynamic stability) among all the four polymorphs.

The energy difference of 9 meV/atom, by which is the formation energy of the polymorph depicted in Figure 1c lower than that of the inverse Heusler structure, is small but significant and more than an order of magnitude above the error bar of our calculations. It should be noted that the concept of mixing atoms in the 2NN shell of Fe${}_{2}$AlCo does not always lead to the lower formation energy. The polymorph, which is depicted in Figure 1d, has this type of mixed 2NN atomic environment in the case of all sublattices within the Heusler structure (all atomic species) and its formation energy is less negative than that of the inverse Heusler variant.

As far as the volume per atom (and equivalently the lattice parameter of the 16-atom supercells) of the two polymorphs with specific mixed occupation of 2NN shells are concerned, their values in Table 1 are quite similar to that of the inverse Heusler phase. A similar statement holds also for the total magnetic moments (per formula unit) but the details of the studied magnetic states are more complicated (see the Section 4 below).

**Figure 2:**
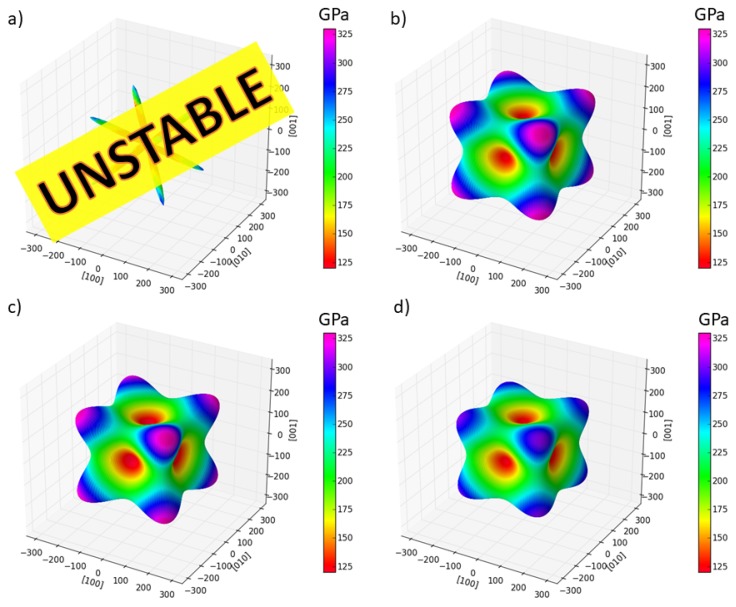
Visualizations of single-crystal elastic properties of Fe${}_{2}$AlCo polymorphs in the form of directional dependences of Young’s modulus for (**a**) the full-Heusler-structure variant, (**b**) inverse-Heusler polymorph, (**c**) specific distribution of Co (and Fe) atoms regarding the atoms in the 2NN shell (see the text), and (**d**) special arrangement of atoms of all three chemical species. The directional dependences were computed from the single-crystal elastic stiffnesses of the studied polymorphs and visualized by the SC-EMA on-line tool [36,37] (http://scema.mpie.de).

Regarding the elastic properties, the single-crystal elastic stiffnesses are listed in Table 1 and visualized as directional dependences of the Young’s modulus in Figure 2. The values of elastic stiffnesses in the case of the full Heusler structure violate one of the conditions of mechanical stability in the case of cubic-symmetry crystals ($C_{11}$ − $C_{12}$ < 0) and, therefore, this phase is mechanically unstable. The other three polymorphs (inverse Heusler and the two variants with special arrangements of atoms) have the values of elastic stiffnesses quite close and, equivalently, the directional dependences of the Young’s modulus in Figure 2b–d are quite similar (the polymorph visualized in Figure 1d being the most different). In all those cases, the <111> and <100> directions are the stiffest and softest, respectively.

**Figure 3:**
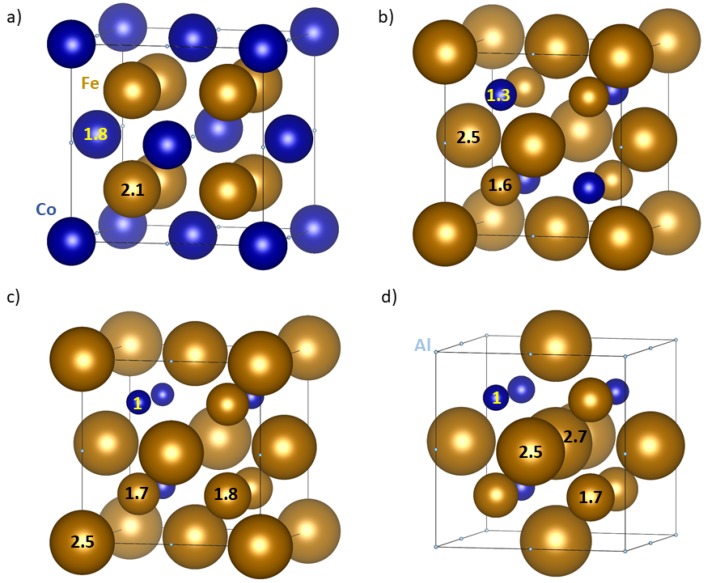
Schematic visualizations of local magnetic moments of atoms in the four studied Fe${}_{2}$AlCo polymorphs in the case of (**a**) the full-Heusler-structure variant, (**b**) inverse-Heusler polymorph, (**c**) specific distribution of Co (and Fe atoms) regarding the atoms in the 2NN shell (see the text), and (**d**) special arrangement of atoms of all three chemical species. Magnetic moments of Al atoms are antiparallel to those of Fe and Co atoms but their magnitudes are so small, less than 0.05 $\mu_{B}$, so that they can be considered as non-magnetic and are not shown in this figure. The numbers within the spheres indicate rounded values of local magnetic moments.

While the total magnetic moments of the four studied polymorphs (listed in Table 1) are quite similar, local magnetic moments of Fe and Co atoms turned out to be all parallel (ferromagnetic states) but very different in magnitude and sensitively depending on the composition of nearest coordination shells. Regarding the full Heusler polymorph, all Fe atoms have the same environment with 4 Co and 4 Al atoms in the 1NN coordination shell and 6 Fe atoms in the 2NN shell. Their local magnetic moments are equal to 2.045 $\mu_{B}$. Co atoms are surrounded by 8 Fe atoms in the 1NN coordination shell and 6 Al atoms in the 2NN shell. Their local magnetic moment is 1.754 $\mu_{B}$ (see schematics in Figure 3a). As far as the inverse Heusler variant is concerned, all Fe atoms have a mixed 1NN shell with 4 Fe atom and then either 4 Al atoms or 4 Co atom. Those Fe atoms with Al in the 1NN shell have 6 Co atoms in the 2NN shell and their magnetic moment is 1.587 $\mu_{B}$ while those with Co in the 1NN shell have 6 Al in the 2NN shell and much higher magnetic moments of 2.528 $\mu_{B}$ (see Figure 3b). Co atoms have 4 Al and 4 Fe atoms in the 1NN coordination shell, 6 Fe atoms in the 2NN and their moment is 1.036 $\mu_{B}$ (see also Figure 3b).

The Fe${}_{2}$AlCo polymorph with one sublattice containing only Fe and Al atoms as in the inverse Heusler structure and the other sublattice with a special arrangement of Co and Fe atoms (Figure 1c) has more non-equivalent magnetic atoms than the Heusler and inverse Heusler variants. Consequently, their local magnetic moments are more complicated. The four Fe atoms on the inverse-Heusler sublattice have 4 Co atoms and 4 Al atoms (specifically arranged) in the 1NN shell and 6 Al atoms in the 2NN shell. All these Fe atoms have their magnetic moment very similar and close to 2.52 $\mu_{B}$. The Co atoms have the magnetic moments equal to 0.91, 0.96, 0.97 and 1.01 $\mu_{B}$ with the Co* atom exhibiting the lowest value. The Fe atoms sharing the sublattice with the Co atoms have the magnetic moments equal to 1.65, 1.65, 1.66 and 1.83 with the Fe* atoms having the highest magnitude (see Figure 3c).

The highest number of non-equivalent atoms is found in the Fe${}_{2}$AlCo polymorph depicted in Figure 1d. The sublattice containing only Al and Fe atoms has one atom Fe*’ surrounded by a special arrangement of 4 Co and 4 Fe atoms in the 1NN shell and 6 Fe atoms in the 2 NN shell. These particular Fe*’ atoms have the highest magnetic moment of 2.65 $\mu_{B}$ but the magnetic moments of other three Fe atoms are also high, 2.50, 2.51 and 2.62 $\mu_{B}$ (see Figure 3d). Co atoms on the other sublattice have the local magnetic moments of 0.95, 0.96, 1.00 and 1.01 $\mu_{B}$ with the Co* atom exhibiting the highest value. The Fe atoms on this sublattice have magnetic moments of 1.63, 1.63, 1.65 and 1.75 $\mu_{B}$ with the Fe* atoms possessing the highest value.

## 4. Discussion

**Figure 4:**
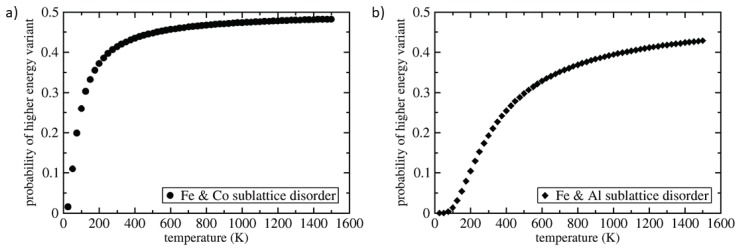
Estimated probabilities of occurrence of higher-energy polymorphs employing Boltzmann statistics: (**a**) for the inverse Heusler phase and (**b**) for the polymorph depicted in Figure 1d.

The energy difference of 9 meV/atom between the polymorph depicted in Figure 1c and the inverse Heusler variant is indeed rather small. Considering the former as the *T* = 0 K ground state and the inverse Heusler polymorph as an excited state, we can estimate the probability of occurrence of the inverse Heusler structure for elevated temperatures using Boltzmann statistics. The results are visualized in Figure 4a. The probability of the occurrence of the inverse Heusler polymorph is close to 0.4 already at the room temperature and it is nearly equal as that of the lowest-energy polymorph at elevated temperatures (structural multiplicity). As these two polymorphs have the same sublattice with solely Fe and Al atoms but different atomic arrangements in the case of the sublattice containing Fe and Co atoms, this structural multiplicity may lead to a chemical disorder on the sublattice containing Co and Fe atoms or co-existence of both phases.

Similarly, the energy difference between the lowest-energy polymorph (Figure 1c) and that depicted in Figure 1d is only 37 meV/atom. Applying the Boltzmann statistics to this pair of polymorphs we got the probability of occurrence of the polymorph shown in Figure 1d visualized in Figure 4b. The probability is about 0.2 for the room temperature and above 0.4 for temperatures above 1075 K. Both these structures have the same atomic arrangement in the case of the sublattice containing solely Fe and Co atoms but differ as far as the sublattice containing Fe and Al is concerned. Again, this structural multiplicity will likely lead to either a chemical disorder on the Fe and Al sublattice or co-existence of both phases.

Our analysis indicated that Fe${}_{2}$AlCo at elevated temperatures may either contain two disordered sublattices (each with different chemical composition) or be formed by different co-existing phases. The first alternative is matching high-temperature experimental data which identify the Fe${}_{2}$AlCo compound to crystallize in the B2-like (CsCl-type) structure, see, e.g., Refs. [38,39,40]. It means that there are two disordered sublattices with different chemical composition but no evidence of more complicated crystal phases with a longer range periodicity is detected. The disorder would also solve the issue related to the fact that the lowest-energy polymorph does not have a cubic symmetry (it is trigonal). On the other hand, in the case of synthesized nano-particles different phases co-exist [41].

Next, inspecting further the values from Table 1, it is clear that the thermodynamically least stable polymorph (with the least negative formation energy $E_{f}$) with the full Heusler structure has the lowest value of the bulk modulus *B* indicating its lower mechanical stability. Reciprocally, the thermodynamically most stable polymorph (shown in Figure 1c) with the most negative formation energy has the second highest value of the bulk modulus as an indication of its excellent mechanical stability (within the four studied variants). This fact is in line with the link between mechanical and thermodynamic stability identified also in other systems when thermodynamically less stable phases possess also lower mechanical stability. This trend was shown in the case of Ti-Nb bcc alloys [42], different polymorphs of Ni${}_{4}$N [43], fcc alloys [44] or in the case of Σ5(210) grain-boundary interface states with different chemical compositions in Ni${}_{3}$(Al,Si) intermetallics [45].

From Table 1 we may see another interesting relation between the magnetic moment and the volume. The highest magnetic moments (in Table 1 listed per formula unit) are obtained for polymorphs with the highest volume per atom. The magnetic moment thus grows with increasing volume (so-called magneto-volumetric argument).

The level of correlation or anti-correlation can be mathematically quantified by evaluating the sample correlation coefficient *r*. In general, for two data sets $f_{i}$ and $g_{i}$, this coefficient is defined as

$$r=\frac{n\sum_{i=1}^{n}f_{i}g_{i}-\sum_{i=1}^{n}f_{i}\sum_{i=1}^{n}g_{i}}{\sqrt{n\sum_{i=1}^{n}f_{i}^{2}-{\left(\sum_{i=1}^{n}f_{i}\right)}^{2}}\sqrt{n\sum_{i=1}^{n}g_{i}^{2}-{\left(\sum_{i=1}^{n}g_{i}\right)}^{2}}}.$$

**Figure 5:**
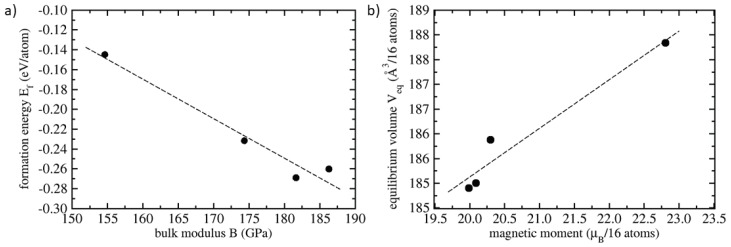
Relations between calculated properties of the four studied Fe${}_{2}$AlCo polymorphs, (**a**) anti-correlation of the formation energy $E_{f}$ and the bulk modulus *B* and (**b**) correlation between the equilibrium volume and the magnetic moment of 16-atom supercells visualized in Figure 1. The dashed lines are linear fits obtained by the least-square method.

If $f_{i}$ and $g_{i}$ are strongly correlated or anti-correlated, the coefficient approaches 1 or −1, respectively. Considering the results for the four studied Fe${}_{2}$AlCo polymorphs summarized in Table 1, we obtain the sample correlation coefficient *r* = −0.979 for the formation energy and the bulk modulus indicating a strong anti-correlation between these two quantities and *r* = 0.972 for the magnetic moment and the volume indicating that they are strongly correlated. Both trends are shown in Figure 5.

Lastly, when considering all the computed results summarized in Table 1, we may note that the full Heusler polymorph of Fe${}_{2}$AlCo is very different from the other three structural variants. This fact is most clearly demonstrated on its mechanical instability (when all other three polymorphs are mechanically stable). We next analyze this mechanical instability. The condition which is violated, $C_{11}$ − $C_{12}$ < 0, is related to a tetragonal deformation and the energy is lowered when the crystal shape is changed to a tetragonal. As the full Heusler structure has the full cubic symmetry the instability is identical along all three axes (cubic-cell vectors). We will simulate such a deformation along the *z* axis.

**Figure 6:**
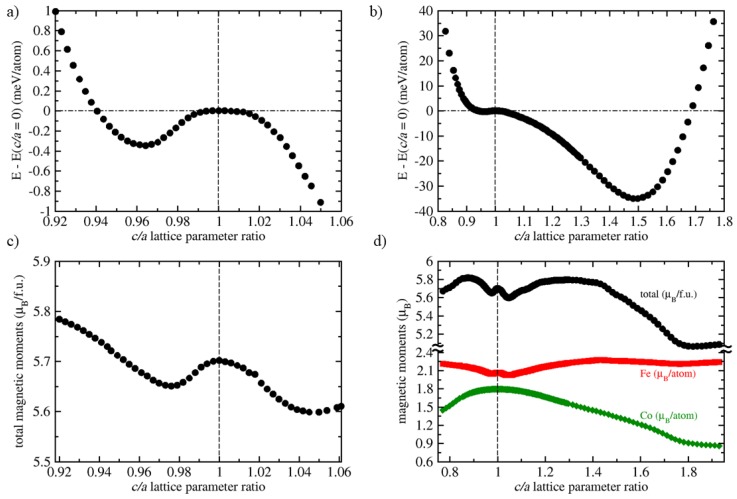
Computed energies and magnetic moments of the full Heusler polymorph of Fe${}_{2}$AlCo transformed along the Bain’s path: (**a**) energies close to the undeformed state (c/a = 1) with a shallow minimum, (**b**) energies for a broader range of c/a values with a deeper minimum, (**c**) total magnetic moment per formula unit for states close to the undeformed state, (**d**) total magnetic moment and local magnetic moments of Fe and Co atoms for a broader range of c/a values. The energies in subfigures (**a,b**) are depicted with respect to the energy of undeformed state with c/a = 1 (indicated by vertical dashed lines and horizontal dash-dot lines). Mind the interruption of the vertical axes in part (**d**).

The simulated transformation path is so-called Bain’s path (see, e.g., Refs. [46,47,48,49,50,51,52]). It is volume-conserving and we will use the equilibrium volume obtained for the full Heusler polymorph. The Bain’s path is conveniently described by the c/a ratio of the lattice parameters with the *c* parameter being that along which lattice is deformed while the *a* is the lattice parameter in the two directions perpendicular to the *c* which are changed so as to preserve the volume. The calculated results are visualized in Figure 6. For small deformations (values of c/a close to 1), the energy exhibits indeed a maximum in line with the earlier found mechanical instability (see Figure 6a). The fact that there is an extreme for the undeformed state is also in line with the theory of symmetry-dictated extrema [46,53,54,55,56,57,58,59] as the c/a = 1 state is the only higher-symmetry state along the Bain’s path in the case of the full Heusler phase of Fe${}_{2}$AlCo. For c/a < 1, there is a very shallow energy minimum (the lowest-energy computed state in this region of deformations has c/a = 0.9642 and the energy lower by 0.35 meV/atom than the undeformed state with c/a = 1). The energy then increases for yet smaller values of c/a. As far as c/a > 1 are concerned, there is a deeper minimum for much larger deformations. The lowest-energy computed state has c/a = 1.499 and energy lower than the undeformed state by 34.95 meV/atom (see Figure 6b). The total energy then increases for yet higher values of c/a.

Regarding the magnetic properties of tetragonally deformed full Heusler phase, the total magnetic moment per formula unit has a maximum for the undeformed state with c/a = 1 and two shallow minima for c/a ≈ 0.92 and 1.05 (see Figure 6c). It is worth noting that the former minimum of the magnetic moment does not correspond to the state with the shallow energy minimum shown in Figure 6a. For a broader range of deformations (see Figure 6d) the magnetic moment decreases for c/a < 0.9 and from c/a ≈ 1.3 till c/a ≈ 1.8. Then it increases again for yet higher values of c/a.

Interestingly, computed values of local magnetic moments of Fe and Co atoms show that Fe atoms (red symbols in Figure 6d) exhibit a maximum for the undeformed state with c/a = 1 as well as the two minima for c/a about 0.92 and 1.05, i.e., for the same states as for which the total magnetic moment has the same type of extrema (black symbols in Figure 6d). Co atoms (green symbols in Figure 6d) do not show any minima of their local magnetic moment for c/a about 0.92 and 1.05 but do have a maximum for the undeformed state with c/a = 1.

## 5. Conclusions

We have used quantum-mechanical calculations to study material properties of four different polymorphs of Fe${}_{2}$AlCo. Two of them were the full and inverse Heusler structures but we have also simulated two other structural variants with a very specific distribution of atoms when Co atoms have on average 3 Fe and 3 Co atoms in their second nearest neighbor shell. This atomic arrangement, which does not appear in either full Heusler or inverse Heusler structure, was suggested by Glover et al. [24] based on their X-ray and Moessbauer data. The lowest formation energy (and thus the highest thermodynamic stability) turns out to be indeed predicted for one of these two polymorphs with this special arrangement of atoms, in particular for that one which has one sublattice (occupied only by Fe and Co atoms) as in the inverse Heusler structure. Due to the fact that this thermodynamic preference is based on very small energy differences (only 9 and 37 meV/atom with respect to the lowest-energy polymorph), we predict the occurrence of structural multiplicity at elevated temperatures, in particular chemical disorder at different sublattices which is indeed seen is experiments.

Next, having the results for all four studied polymorphs, we can conclude that the total magnetic moment is clearly correlated with the volume, the states are ferromagnetic in their nature but the local magnetic moments of Fe and Co atoms within different polymorphs can become very complex and sensitively depend on the chemical composition of the first and second nearest neighbor coordination shells. Lastly, when evaluating the mechanical stability by computing anisotropic single-crystal elastic stiffnesses, we have found that there is a link between thermodynamic and mechanical stability. Thermodynamically more stable compounds tend to have higher value of the bulk modulus and, for example, the full Heusler polymorph of Fe${}_{2}$AlCo is not mechanically stable with respect to the tetragonal transformation (Bain’s path). Further studies, both experimental and theoretical, of this interesting issue would be very desirable.

## Figures and Tables

**Table 1:** Calculated thermodynamic, structural, magnetic and elastic parameters of the studied Fe${}_{2}$AlCo polymorphs. Listed are values of the formation energy $E_{f}$, equilibrium volume per atom $V_{\mathrm{eq}}$, equilibrium lattice parameter $a_{\mathrm{eq}}$ of 16-atom supercells shown in Figure 1, magnetic moments $\mu^{\mathrm{TOT}}$ per formula unit (f.u.), and single-crystal elastic stiffnesses $C_{11}$, $C_{12}$ and $C_{44}$ together with the bulk moduli *B* derived from these elastic stiffnesses (*B* = ($C_{11}$ + 2$C_{12}$)/3).

Polymorph	*E*${}_{f}$	*V*${}_{\mathrm{eq}}$	*a*${}_{\mathrm{eq}}$	$\mu^{\mathrm{TOT}}$	*C*${}_{11}$	*C*${}_{12}$	*C*${}_{44}$	*B*
	(eV/atom)	(Å${}^{3}$/atom)	(Å)	($\mu_{B}$/f.u.)	(GPa)	(GPa)	(GPa)	(GPa)
full Heusler	−0.145	11.740	5.727	5.70	148	158	115	155
inverse Heusler	−0.260	11.556	5.697	4.99(8)	245	157	139	186
Co & Fe with mixed 2NN	−0.269	11.562	5.698	5.02	235	155	139	182
Co, Fe & Al with mixed 2NN	−0.232	11.617	5.707	5.08	233	145	125	174
